# Construction of Hospitals for the Insane

**Published:** 1884-04

**Authors:** J. C. Spray

**Affiliations:** Superintendent Cook County Hospital for Insane


### ARTICLE V.

CONSTRUCTION OF HOSPITALS FOR THE INSANE. By Dr. J. C. SPRAY, Superintendent Cook County Hospital for Insane.

It may be laid down as a generally accepted proposition, that an institution for the insane is an hospital, and not a mere place of custody. The latter function was for a long time looked upon as the chief function of institutions for the insane, and as a consequence structures modeled on prisons have been erected at a large cost, which are worse than useless for the purposes of hospitals for the insane. Since an institution for the insane is an hospital, and treatment is the object of an hospital, everything in its management is only a means toward that end.

Into the treatment of both the sane and insane enters not only the question of medicines and diet, but the influence of impressions made on the mind. As Bucknill and Tuke * have said, "Dyspepsia caused by anxiety is cured by prosperity and content; the dysentery of armies waits upon the depression of defeat, and is cured by the breath of victory." As mental impressions are derived from one's surroundings, it follows that everything connected with an hospital for the insane should be made subservient to the one purpose of treating the inmates, since it has been found by extensive experience that treatment of the insane in specially devised hospitals yields the best results. The patient, who at home is the center of well meant but illy directed sympathy, and in consequence the monarch of all he surveys, becomes, on entering an hospital for the insane, one of the members of a large community, whose surroundings demonstrate to them that they are regarded as sick people, whether they so regard themselves or not. The mind of the insane is so constituted that it can originate nothing but what is tainted by disease. Healthy ideas may be introduced from without, which are stronger in their influence than those originating in the mind of the patient. To introduce healthy ideas, the manner adopted must be such as will not arouse opposition; the healthy ideas must be insinuated into the mind. If the idea be insinuated into the patient's mind by his surroundings that he is regarded as a person to be treated in an hospital for his sickness—insanity—this idea tends to develop self-control, and the development of self-control tends to cure or alleviate insanity. Cases are met with every day of patients treated at home and in an hospital for the insane with exactly the same medicines. Those treated at home, as a rule, die or become permanently insane; the others as

* Psychological Medicine.

frequently recover. Spitzka†, an impartial authority of repute, says: "An hospital sojourn has, in the vast majority of cases, a good effect on the insane. Curable cases are never injured in their prospects in a medically well-managed institution, and incurable cases should be there for practical reasons, and are better off in than out of an asylum. The advantages of hospital treatment are these: refusal of food and medicines, great obstacles to the treatment of the insane outside of hospitals, are best dealt with by a skillful corps of physicians and attendants always on the spot, with the requisite appliances at their disposal. The necessary supervision of the insane at all hours can be carried on at least expense and greatest thoroughness in the hospital ward. The excessive use of narcotics, and restraint necessary for preventing scandal, noise, and destructiveness at home are not required in the hospital. The sojourn of a patient in a hospital for the insane, and the restraint of its walls, is a constant reminder that he is regarded as insane. It is, in many cases, a far stronger incentive to a kind of reflection which leads to the correction of delusions than any drug."

It is clear then that the greatest effect of hospital treatment is due to the impression made on the mind of the insane patient. If the hospital for the insane be built like a prison; if it be conducted more on the idea of saving money out of the patients than of saving money through curing them, it will damage its patients as much as it would otherwise benefit them. When hospitals for the insane are so built as to impress the patient with the belief that he is entering a prison, such an impression has a very bad effect on him, which tends to make his disease resist treatment otherwise beneficial. If, however, the hospital be built in such a manner as makes the first impression agreeable, the patient is, despite himself, favorably impressed, and this favorable impression is strengthened or diminished as the interior structure or management most resemble those of an hospital or a prison. In the hospitals of the eighteenth century, which were constructed on a prison plan, the insane displayed a fury which would be scarcely credible, were it not that similar pictures are presented from almshouses where the insane, being

†Insanity; its Classification, Diagnosis and Treatment.

few in number, are shackled and confined in dungeons. Dr. A. Bennett*, of the Norristown, Pennsylvania, Hospital for the Insane, has had under observation a patient who had been chained in a dungeon, and her food thrust into her den through an opening in the wall, which she must eat as best she could with her hands tied. This patient, a tall, powerful German woman, came to the Norristown Hospital with a horrible reputation for ferocity. In a very short time she became tractable and quiet, and presented an appearance calculated to attract rather than repel. Being asked, why she was so wild in the almshouse and so quiet in the hospital, she replied : " Because they locked me up like a beast. How would you like to be locked up like a beast ? " From this case, which could be duplicated thousands of times in the history of the English, French, Italian, German, and other European insane in the eighteenth century, it can readily be seen that the impression made on the patient by the external appearance and internal appointments of an hospital, is a very important element in the treatment. It may, therefore, be accepted as a fact settled by years of experience in England, Ireland, Scotland, Germany, France, Italy, Holland, and the Scandinavian countries, that the external appearance of an hospital should be the most cheerful and alluring possible.

The cell-like construction of many asylums was due to the fact that they were modeled on old monasteries. These cells were intended to secure for pious monks, the very thing it is desirable for the insane to avoid, the opportunity for unwatched solitude. The insane patient shut up by himself alone or with a single companion finds his disordered fancies exercising complete sway over his mind, while the presence of a single companion tends to aggravate these and excite him to violence. The insane patient under such circumstances readily conceives that he is looked upon as a criminal, and this causes either deep dejection or great violence, as the patient is inclined to be sad or furious. If the last, he has combative tendencies aroused by a fancied infringement on his rights, and violent deeds are not unfrequent under such circumstances. It is a matter of every day experience that men

---

* Journal of the Medico-Legal Society, July, 1883.

who are violent and uncontrollable in an hospital built on the cell plan quiet down and become the most tractable patients of an hospital built on an hospital plan.

The internal construction of an hospital for the insane is, therefore, of no less importance than its external appearance. It is therefore necessary to inquire what system of internal construction has been found to answer best. Prominent among those active in solving the problems of hospital construction for the insane, have been the physicians of England, Ireland, and Scotland. And as Dr. P. M. Wise,* of the Willard Asylum for the chronic insane, New York, remarks, the first thing which strikes a visitor to the institutions in those countries is that in the county hospital for the insane, the single room accommodation rarely exceeds 20 per cent. of the whole. The associated dormitories frequently contain fifty beds or more, with a few single rooms attached for exigencies. Associated dormitories, it appears, are the prominent feature of the hospitals for the insane of countries where most progress has been made in the treatment of the insane.

Dr. Lalor,† of the Richmond District Lunatic Asylum, Dublin, Ireland, after an extended trial of a few associated dormitories, recommended that a "number of long, narrow, unsocial corridors and cheerless single rooms, be converted into large day and sleeping rooms." As a result of this, "far superior means of supervision, of occupation, and of amusement were afforded, while the accommodation for the insane was greatly increased at a small cost." Of these associated dormitories, Dr. R. Boyd,† a leading English authority on construction of hospitals for the insane, says: "The abolition of single rooms and cells and the substitution of associated dormitories, is a great improvement, as it does away with the prison-like appearance, which has a depressing effect on those mentally afflicted."

Dr. G. F. Blandford,‡ another prominent English authority, says: "The abolition of solitary cells and the association of patients by night and day, also conduces greatly to their quiet and

† *Journal of Mental Science*, Vol. XVI.

*Journal of Mental Science*, Vol. XXIII.

* *Alienist and Neurologist*, Oct. 1882.

† Reports for 1878-1882.

well-being," and this is also the opinion of Dr. Mackintosh,|| a prominent Scotch authority. Dr. Wise cites these associated dormitories as one of the features in which English, Irish, and Scotch asylums compare favorably with those of the United States. Dr. Pelman,¶ a leading German authority, bears equally strong testimony to that of Dr. Boyd, and the changes introduced into France, Italy, and the Scandinavian countries are more or less modeled on the examples set by the asylums of Great Britain and Ireland. It may, therefore, be accepted as settled by European experience that hospitals constructed with extensive associate dormitories are best adapted for the benefit of the patients. There are certain objections which are likely to arise as to this dormitory system. At first it would seem as if a greater amount of restraint would be rendered necessary, but in the countries where the system is most followed, in Ireland, England, and Scotland, restraint is reduced to the greatest possible minimum.

Second. The question of adaptation to American hospitals would naturally arise. It does not follow because a thing has been found practicable in Europe that the same will be successful in America.

With regard to this question, Dr. Wardner ** who has had an unwilling experience, caused by the fire in his hospital for the insane at Anna, Ill., says: "These patients have been contented, and the number of escapes has not been as many as in former years. Sleeping in such large associate dormitories seems to have had the effect of keeping them quieter, and with the exception of occasional excitement from epileptic attacks there has not been as much disturbance as would have occurred with the same number of sane people lodged in the same room. Those who had been seriously noisy and disturbed their neighbors, while occupying single rooms or small dormitories, out of consideration for others or in consequence of the restraining influence of numbers and the eye of the night attendant, became quiet and acquired the habit of keeping still, and of sleeping well. In fact, a general improvement has been observed in both mental and phys-

|| *Journal of Mental Science*, Vol. XVI.
¶ *Journal of Mental Science*, Vol. XVI.
** Alienist and Neurologist, Oct. 1881.

ical health." Similar testimony is given by Dr. Catlett †† of the Hospital for the Insane at St. Joseph, Missouri, who had a very similar experience. Dr. Kiernan, ‡‡ at one time assistant physician of the Ward's Island New York City Hospital for the Insane, states that the overcrowding of that institution led to the use of associated dormitories, which were attended by good effects, and Dr. Godding, §§ now of the Government Hospital for the Insane at Washington, from an experience at the Taunton, Mass., Hospital for the Insane, is of opinion that associated dormitories for seventy-five patients are not attended by any but good results. It is therefore clear that this system is practicable in the United States. The question of cost is another very serious question to be considered. From the experience of Dr. Lalor, already cited, it is evident that an hospital built on the associated dormitory system costs less than one built on the cell plan.

Dr. I. Ray *** accounts for the great discrepancy in cost between American and European hospitals for the insane by this very element of associated dormitories. He says, "the point which may account for comparative cheapness of construction in Great Britain is that a much larger proportion of the patients sleep in associate dormitories. In Scotland this is especially the case. In the pauper hospital for the insane at Morningside, Scotland, with some hundreds of patients there were only a dozen single sleeping rooms." Taking all the circumstances cited into consideration it follows, First: That associate dormitories act in a beneficial way on the insane, and tend to diminish suicides, since these are committed when the patient is alone. Second: That they are perfectly well adapted for use in hospitals for the insane in the United States. Third: That the cost of associate dormitory hospitals is much less than that of cell hospitals.

If associate dormitories are, for the reasons cited, best adapted to the treatment of the insane, the question of rendering effective these and other agencies in the treatment of the insane nat-

†† Report for 1881.

‡‡ Lectures on Insanity.

§§ Cited by Kiernan Op. cit.

*** Journal of Nervous and Mental Disease 1878.

urally arises. Among the elements which enter into this problem are, the selection and number of attendants. In the New York City hospitals for the insane, a proportion of one attendant to 14 patients has been found to be the lowest number compatible with the proper employment and care of the insane. In the county asylums of Great Britain and Ireland, the preferable ratio has been found to be one attendant to every 11 patients, and it should be remembered that on account of the almost universal adoption of associate dormitories, this proportion of attendants is really much more effective than it would otherwise be since one night attendant, under the associate dormitory system could better supervise 75 patients than one attendant could five under the cell system.

Owing to this large proportion of attendants, restraint in the English asylums is reduced to a very minimal amount, and the increased cost of attendants is very well made up by the decreased opportunities of the patients for destructiveness. If such were not the case, the English rate-payers, who are the governing body of the English county hospitals, and who are by no means inclined to liberality, since they pay for the support of these institutions, would not sanction the employment of so large a number. From the higher standpoint of humanity, the contact of many sane minds, free from the trammels of kindred and under medical direction, with the insane is very desirable, since it tends to introduce sane ideas into minds which are incapable of originating these for themselves. The insane patient readily perceives, as a rule, the insanity of his associates. A curious instance of this kind is narrated by Dr. Spitzka.††† In consequence of his attempts to alleviate the condition of the insane in New York, he became known as the friend of the insane. The courts discharged from hospitals for the insane several undoubted lunatics as "sane." Each of these admitted to Dr. Spitzka that every one of those liberated, except the one making the communication, was a lunatic. The fact that others who claim to be sane, are justly treated as lunatics, naturally leads the insane person to look upon his mental operations with suspicion at times. If he have too few sane minds in constant contact with him, he is, while benefited, rendered somewhat

†††*American Journal of Neurology and Psychiatry*, Vol. II.

secretive, and this secretiveness retards a recovery which would be accelerated by contact with sane minds properly trained for contact with the insane. In the English, Irish, and Scotch hospitals for the insane, not to speak of the German, Italian, French, Scandinavian, and Swiss institutions which follow in the wake, attacks of furious excitement are neither so frequent nor so prolonged as in the American institutions scantily provided with attendants.

This fact is of great significance. Under circumstances which it might be imagined would aggravate the disease, it is alleviated. It is important in this connection to ascertain the qualifications of English attendants. According to Bucknill and Tuke*: "A good attendant ought to be a good observer, to have the natural power of remarking differences between one patient and another, and in the same patient at different times; keen to notice circumstances which distress and those which soothe, apt to remark all signs of disturbance and danger, and all means of influence...... Mere apathetic good humor is a negative quality, if not a disadvantage. An attendant on the other hand should not be fussy, but should gain and maintain influence by constant care and kindness." With attendants in the ratio mentioned and chosen for their possession of the qualities cited, it is not at all astonishing that the English should be able to do so much for their patients, and that the types of insanity frequent in the United States should present somewhat different features, since the entire management of the hospital aims at the treatment of the patients.

It is obvious that in addition to the great frequency of single rooms causing disadvantages, the American asylums have too few attendants. That large associated dormitories in the asylums, containing from fifty to seventy-five patients, should have one night attendant is self-evident. It is also clear that during the day the patients should be brought into contact with as many attendants as possible, since provision for no more attendants than can do routine duties prevents attendants exercising the proper control over their patients, prevents them from inducing the latter to labor, and also renders necessary more restraint than is

* *Psychological Medicine.*

desirable. An overworked attendant can neither be as cheerful nor as disposed to make allowances for the outbreaks of the patients under care as is desirable for their proper treatment; there can be but little doubt that many of the cruelties perpetrated on the insane are perpetrated in institutions too scantily supplied with a sufficient staff of attendants. In the English, Irish, and Scotch institutions attendants much more rarely perpetrate cruelties than in the institutions of the United States, and in more than one instance an explanation of the cruelty is to be found in the fact that the attendant has been left alone to manage too many patients for too many successive hours. A large staff of attendants implies more labor on the part of the patients, less destructiveness, less chance for cruelty, and inferentially a greater number of recoveries. The English ratio of attendants to patients is therefore a desirable one. A question arising out of the construction of associated dormitories is that of how many small rooms shall be provided for exigencies in as close proximity as practicable, provisions being made for the exclusion of sound. There are no very certain datas to serve as a guide, but in some American institutions three single rooms to each associated dormitory of twenty-five patients, has not been found to be an excessive proportion. Judging from this, a proportion of nine single rooms to each associate domitory of seventy-five beds is desirable to meet all possible emergencies.

The important elements in the construction and internal management of an hospital for the insane, then, are: First: A cheerful external appearance; to secure this it would be desirable not to use bars, but gratings on the interior and not exterior of windows. The windows should be large paned, so as to afford as much light as possible. These are less expensive in the end than small panes, and give a much more cheerful appearance. Second: Associate dormitories, with single room provision for about twenty per cent. of the patients, inclusive of the rooms annexed to the associate dormitories. The capacity of the associate dormitories should be about seventy-five, and these associate dormitories should be separate from the day rooms. Dining rooms should be provided with a capacity for one-half or the whole inmates of the ward. Third: Heating of the institution should be by direct radiation. Fourth: The building should be lighted by gas, as being the most cleanly, and by experience, the least dangerous. Fifth: The number of attendants should be as near the English ratio of one attendant to eleven patients as possible.

